# The Role of Proton Transfer on Mutations

**DOI:** 10.3389/fchem.2019.00536

**Published:** 2019-08-21

**Authors:** Ruby Srivastava

**Affiliations:** Bioinformatics, CSIR-CCMB, Hyderabad, India

**Keywords:** proton transfer, mutations, tautomerism, quantum tunneling, oriental polarization

## Abstract

Hydrogen bonds play a critical role in nucleobase studies as they encode genes, map protein structures, provide stability to the base pairs, and are involved in spontaneous and induced mutations. Proton transfer mechanism is a critical phenomenon that is related to the acid–base characteristics of the nucleobases in Watson–Crick base pairs. The energetic and dynamical behavior of the proton can be depicted from these characteristics and their adjustment to the water molecules or the surrounding ions. Further, new pathways open up in which protonated nucleobases are generated by proton transfer from the ionized water molecules and elimination of a hydroxyl radical in this review, the analysis will be focused on understanding the mechanism of untargeted mutations in canonical, wobble, Hoogsteen pairs, and mutagenic tautomers through the non-covalent interactions. Further, rare tautomer formation through the single proton transfer (SPT) and the double proton transfer (DPT), quantum tunneling in nucleobases, radiation-induced bystander effects, role of water in proton transfer (PT) reactions, PT in anticancer drugs–DNA interaction, displacement and oriental polarization, possible models for mutations in DNA, genome instability, and role of proton transfer using kinetic parameters for RNA will be discussed.

## Introduction

**Figure 1:**
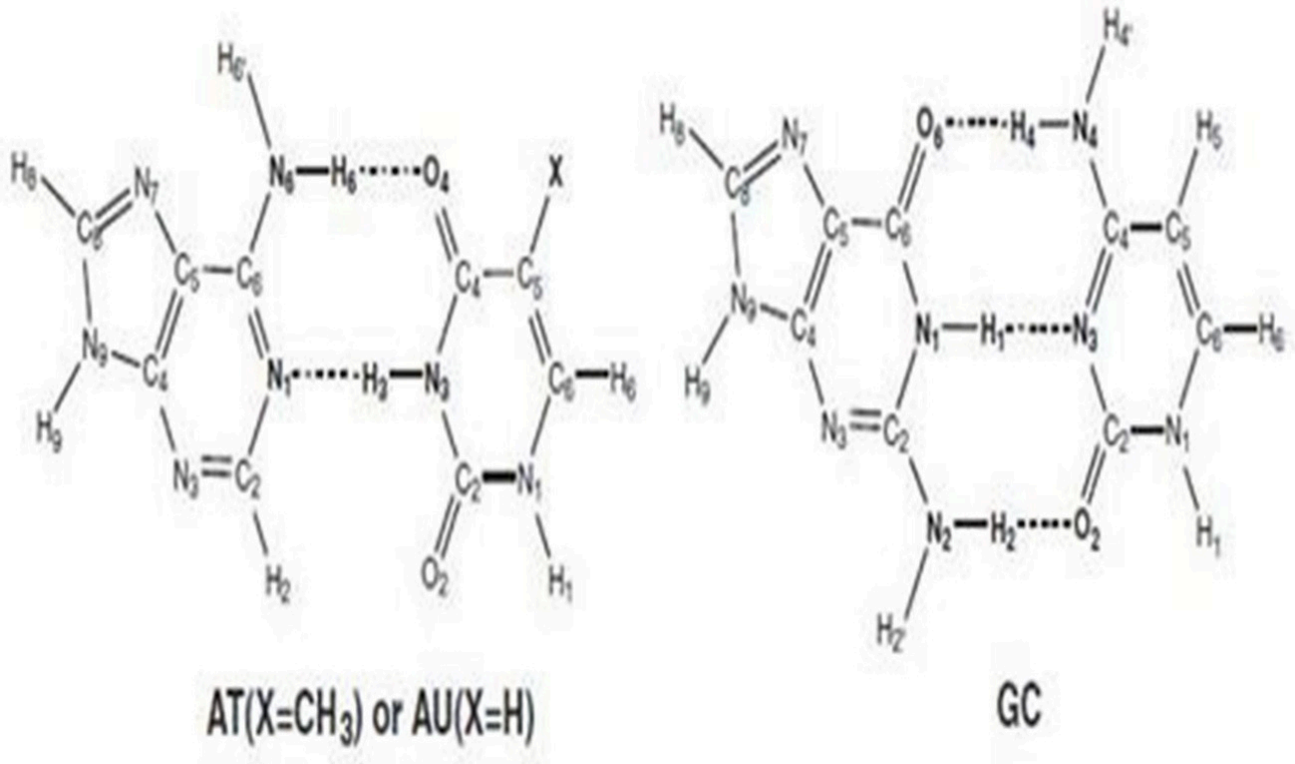
The Schematic representation of DNA and RNA base pairs.

The cellular machinery, which has been managed by nature for millions of years, still shows errors during DNA replication. These errors generate mutations that affect health disorders. Two types of mutations occur during DNA replica. One is the induced mutation, which is caused by the external agents as free radicals or radiations, but can be reduced by prevention of high-risk factors. The other is the spontaneous mutations that are caused by the action of any external element. The origin of these mutations is still unclear. The protons change their positions during the interchanging of interbase hydrogen bonding in DNA bases. The process occurs due to unwinding of DNA strands during proton transfer (PT) reactions with the formation of “rare tautomers.” Rare tautomers promote the spontaneous mutations and are not detected easily. These mutations are not referred to the random mutations in Watson–Crick (WC) pairs [the genetic code being located in the guanine–cytosine (GC) bases]. The position of SPT or DPT differs in these tautomers. As the detection of these tautomers is difficult, computational approaches are applied to study the PT reactions and its biological consequences in DNA and RNA nucleobases. PT mechanism is defined by the movement of a proton (H+) from one molecule to another. These reactions play an important role in acid/base chemistry, heterogeneous and homogeneous catalysis, corrosion, proton exchange membrane fuel cells, and biochemical processes. The DNA consists of adenine (A), guanine (G), cytosine (C), and thymine (T), while in RNA, thymine is replaced by uracil (U), with the other bases remaining the same. The schematic representation of the structures is given in Figure 1. Thus, RNA gives the corresponding base pairs as AU, and GC. Theoretical studies predict that AU is energetically more stable than AT. Sixty-four possible triplet codons are translated into defined sequence of 20 amino acids linked via peptide bonds.

During tautomeric equilibrium, the interbase hydrogen bonds are transferred as positively charged (proton) carriers. Proton transfer in hydrogen bonding also involves many protons, and these multiproton transfers occur in a planned or stepwise manner between the acid–base sites. These bonds act as the stabilizing factor for the DNA duplexes, even when there are stacking interactions between the bases. The hydrogen bonds join the “stair-step” base pairs in DNA duplexes. The strength of hydrogen bond is important to control the replication and transcription process during cell division along with the other controlling factors as solvent, counter ions, hydrophobicity, local dielectric of surrounding water, pH, pK_a_, and temperature.

**Figure 2:**
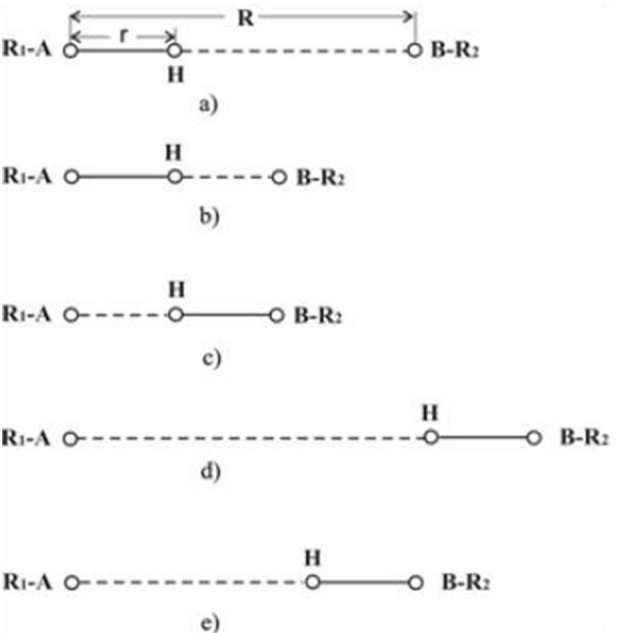
Diagrammatic representation of hydrogen bonding between R1-A and B-R2.

In natural and synthetic catalysis, the transfer of a single proton is difficult due to the size of the catalysts and the complex multi-step or multi component nature of the reactions. The proton transfer reaction involves three basic steps: (a) hydrogen bonding of the acid (A-H+) site to the base (B) forming intermediate A-H+…B complex, (b) movement of proton from A to B, and, finally, (c) dissociation of A and B-H+. Proton transfer reactions occur rapidly, with diffusion being the slowest with a lifetime of 1 × 10^−10^ s (see Figure 2).

**Figure 3:**
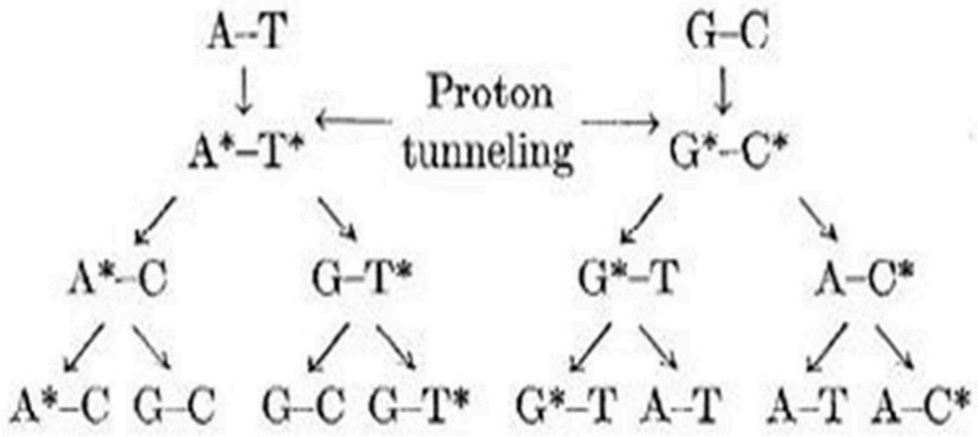
Schematic representation of various possible rare tautomeric states of Watson–Crick (WC) base pairs.

The most interesting feature of these PT reactions is the unexpected results of the experiments and computational calculations given for the studied structures. The stabilization of intermediates also has a vital role in mutagenic studies of these nucleobases apart from the other factors. The critical steps of mutations involve intra- and intermolecular tautomerizations (Löwdin, 1963; Sevilla et al., 1995). The tautomers are important in medicinal chemistry, drug design, and chemical information systems mostly for the organic and bioorganic reactions (Miller et al., 1994; Armitage, 1998; Burrows and Muller, 1998; Boudaïffa et al., 2000) (see Figure 3). DPT is the possible path for mutations with ions, radicals, or surrounding molecules as the supporting factors. The error introduced in formation of rare tautomers during PT reactions affects the important biochemistry processes as diseases.

## Quantum Tunneling in DNA

**Figure 4:**
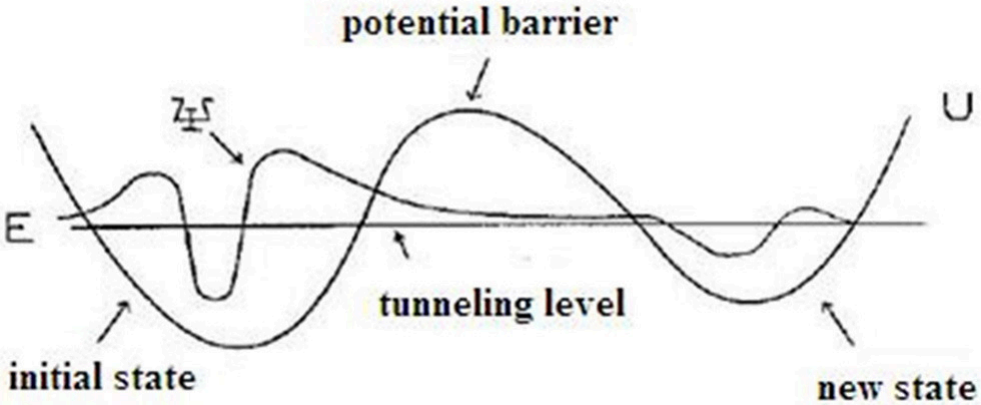
Diagrammatic representation of quantum tunneling effect in quantized wave packet.

In this section, we will discuss the occurrence of proton tunneling, the effect of radiation on the frequency of proton tunneling, and its result on DNA replication integrity. DNA is used as an information storage biological species as the encoded double-stranded helix structure of nucleobases contain the necessary genetic information with the mirror copy of the other strand (Lodish et al., 2000). This recorded information is just like the basic unit of information (bits) on the magnetic tape, i.e., the data storage system. The encoded messages are transferred to the ribosome during protein synthesis, which transforms it into the receipts of protein assembling. During the replication process, the unzipped DNA base pairs are processed by one of DNA-polymerase (DNA-pol) enzymes. All DNA-pols have intricate geometrical structures. The second strand is assembled by the required information and a new floating deoxynucleotide triphosphates (dNTP) molecule is constructed. The performance of DNA and RNA during the transfer of genetic information is described by the computational algorithms. The dNTP selections that use the quantum tunneling of protons as the main dynamical mechanism require the multistep quantum process. The quantum tunneling effect of quantized wave packet is shown in Figure 4.

Hydrogen bonding occurs by the sharing of a single proton between two lone electrons (electronegative atoms N, O) in nucleobases. The two separate atoms contain an extra unpaired electron (outer orbital shell) to possess the single proton. In tautomeric forms, a proton is moved from its original lone pair to another position. The genetic code is affected when the changes occur from normal to the tautomeric forms, generating errors in DNA replication, and causing mutations. The amplified mutations further lead to severe mutations. In DNA, huge competition occurs to catch the protons in the surrounding environment by the multiple electron lone pairs of several molecules. The single proton–electron system is modeled as a single-well potential. The double-well potential is formed with bumps or potential barrier, when two lone pair electrons compete for a single proton in the hydrogen bonding, which are represented as equilibrium shifts in the following form.

N: H                            : N N:                           H: N

The protons opt tunneling (quantum jump) from one equilibrium state to another through the potential barrier. Protons move in the classically forbidden regions to produce the oscillations with a non-stationary state, extended to symmetric and an asymmetric double-well potential (Parker and Van Everv, 1971). In DNA structures, a minimum of two hydrogen bonds are always involved in the process. Hence, it can be considered as a quantum mechanical two-body problem. For maximum stability, it was assumed that the double-well-potential is highly asymmetric in DNA duplexes. A large potential barrier is required to reduce the frequency of tautomeric formation and to ensure high purity of DNA replication. The proton tunneling time depends on the form and height of the barrier. Tunneling occurs in both directions through the potential barrier. Reverse tunneling also occurs in two ways either with radiation or through normal proton tunneling. In the ground state of the AT base pair, the lowest tunneling level has a proton lifetime of 0.165 × 10^−2^ s with a corresponding tunneling time of τ_1, 2_ = 0.346 × 10^−4^ s, and the highest tunneling level has a proton lifetime of 0.366 × 10^−2^ s and a tunneling time of τ_1, 2_ = 0.247 × 10^−11^ s.

The influence of quantum tunneling, which has significant contribution on proton transfer and the reaction pathways (Godbeer et al., 2015) was studied for the canonical and tautomeric charge-neutral forms of the adenine–thymine base pair (A–T and A^*^-T^*^, respectively) with the density functional theory (DFT). The conformational and tautomeric composition of monomers of (tetrazol-5-yl)-acetic acid (TAA) (Araujo-Andrade et al., 2014) were also studied with B3LYP/6-311++G (d,p) level using DFT. 2D potential energy was generated at MP2/6-311++G (d,p) level and a proposal was given to study the tautomerization process during sublimation.

In a latest study, the hydrogen bonds and its use in molecular recognition were studied by comparing the tunneling-assisted quantum entanglement shared in the ground states of covalent and hydrogen bonds (Pusuluk et al., 2018). It was seen that there is significant amounts of quantum entanglement for the thermal state of hydrogen bond. The density-functional theory calculations with hybrid functionals and van der Waals corrections, and optimized path-integral ring-polymer methods (Litman et al., 2019) were used to calculate the quantum vibrational spectra and reaction rates for the nuclear quantum dynamics of intramolecular DPT in porphycene. The results indicated that below 100 K, the concerted double hydrogen transfer (DHT) tunneling pathway dominates, while including the nuclear quantum effects for 100 and 300 K, the concerted and stepwise pathways are selected. Further, the researchers provided architecture for the physical understanding of hydrogen transfer dynamics in the complex systems.

## Proton Transfer in Nucleobases

The mutations (transitions) are reversible in which the DNA replicates continuously. Repeated transitions revert back to their original structures. In these mutations, the DNA strand turns either into junk DNA and thus fails to pass on its encoded information and directions, or turns into malfunctioned DNA to give destructive information. In the twentieth century, quantum-mechanical studies were carried out to predict the tautomeric equilibria of heterocyclic compounds. Few parameters such as ultraviolet spectra, dipole moments, and ionization potentials were calculated using semi-empirical or non-empirical quantum-mechanical computational method as per the requirements. These calculations were found to be useful to predict the relative stability of the tautomers in vapor phase in solution and the influence of substituents (Kwiatkowski et al., 1986). The change in tautomeric equilibria of DNA bases that occurs due to the changes from an inert to a polar environment was discussed in a review with the extensive work of the past 14 years (Person et al., 1989). The tautomeric equilibria of 2(4)-mono oxopyrimidines in the gas phase and solution were studied at low-temperature matrices (Nowak et al., 1980). Energies, heats of vaporization, and UV spectra were calculated and compared to the known experimental data. A study on potential energy surface (PES) of guanine performed by Prof. Leszczynski's group showed that the PES of guanine is not flat. The DFT studies were carried out at MP2/6-31G(d,p) reference geometries with MP4(SDTQ)/6-31G(d,p), MP4(SDQ)/6-311G(d,p), and MP2/6-311++G- (df,pd) levels (Leszczynski, 1992, 1998). Further photo-induced proton transfer for 2-(2′-hydroxyphenyl)benzoxazole (HBO) was studied (Ogawa et al., 2000). The basic feature of this model was the study of tautomerization in the biological environment of duplex DNA. Further, it was seen that the biochemical knowledge on biosynthesis of nucleotides, DNA (and RNA) sequence information and transmission, interaction of nucleic acids with proteins, and DNA sequence rearrangements and alterations are the most important things (Blackburn and Gait, 1990). Computational studies about the tautomerism and protonation of guanine and cytosine in the gas phase and in aqueous solution were carried out for the most stable tautomeric forms of the neutral and protonated nucleic acid bases (Colominas et al., 1996). The nature of 10 nucleic acid base stacking was studied at that time by non-empirical *ab initio* and empirical potential (EP) characterization with planar MP2/6-31G^*^ optimized geometries and many-body correction at the HF/MINI-1 level. Base stacking was investigated in six B-DNA and two Z-DNA base pair steps. The sequence-dependent variations of the total base pair step stacking energies range from −9.9 to −14.7 kcal/mol with the adjustment of the standard combination of a Lennard–Jones and atomic point charge terms (Sponer et al., 1996). DFT studies were carried out for 28 H-bonded DNA (formed by A, T, G, C) base pairs at HF/MINI-1 DFT level of theory (Sponer and Hobza, 1994). The results indicated the H-bonded structure of the cytosine dimer to be more stable than the stacked structures of these pairs. Similarly, the double-proton transfer in adenine–thymine (AT) and GC base pairs in gas phase (Gorb et al., 2004) within room temperature was studied with the inclusion of environmental effects using DFT with B3LYP/6-31G(d) and MP2/6-31G(d) level. The results revealed that the hydrogen-bonded bases possess non-planar geometries. The calculations predicted greater stability for canonic or rare forms of the DNA bases occurring in water molecules and metal cations. Several other computational studies were also carried out for the nucleobases (Hobza, 1986; Stewart et al., 1994; Gould et al., 1995; Jarzynski, 1997).

The intramolecular proton transfer in mono- and dihydrated tautomers of guanine (Gorb and Leszczynski, 1998; Gorb et al., 2004) was studied by HF and the MP2 levels of theory with MP4(SDQ)/6-31G(d)//MP2/6-31G(d) and the MP2/6-311++G(d,p)//MP2/6-31G(d) approximations. It was found that the two-fold water influences the NH_2_-non-planarity phenomena and decreases the non-planarity for the oxo-tautomers. The two-fold water is the source of non-planarity for the hydroxo tautomers and it decreases the non-planarity for the oxo tautomers.

Another study was carried out for infrared laser-driven double proton transfer (DPT) by a 2D model (Abdel-Latif and Kühn, 2010) using the stepwise and concerted transfer pathways. It was concluded that the driven wave packet is dependent on the parameters of the model Hamiltonian and the propagation time. The stepwise mechanism was dominated in most of the cases, while concerted transfer via tunneling occurs for high barrier systems. The field of proton transfer in hydrogen-bonded networks was also explained in the review by Marx (2006). The review gave insights into Grotthuss diffusion in water, excited-state proton transfer in solution, phase transitions in ice, and protonated water networks in the membrane protein bacteriorhodopsin with the *ab initio* simulation techniques.

Computational calculations on the hydrogen atom transfer in the cytosine–guanine base pair (Villani, 2010) and its coupling with electronic rearrangement were studied. It was observed that a different behavior occurred when the hydrogen transfer begins with a H of the guanine or of the cytosine and concerted (synchronic in the N–N and asynchronic in the N–O) double-hydrogen transfer can be activated only when the first H atom of guanine is moved. The concerted double-hydrogen process begins with the hydrogen atom of a purinic base. *Ab initio* constrained molecular dynamics and metadynamics were used to study the mechanism of proton transfer in DNA base pairs (AT, GC) in the gas phase at room temperature (Xiao et al., 2012). Interestingly, the results reveal the DPT in the GC base pair to be a concerted and asynchronous mechanism and in AT to be a stepwise and an asynchronous mechanism. The double-proton transfer reactions in WC GC base pairs were studied after the addition of hydrogen atom (Lin et al., 2012) and the structural changes and energy differences were compared to explore the DPT mechanism. The results revealed that the concerted double-proton transfer mechanism is favorable in the gas phase and the stepwise mechanism is favorable in water with the PT products being energetically less favored.

Oxidized nucleobases (removal of one valence electron) also exhibit enhanced acidity, which leads to PT between the strands of DNA (Ghosh and Schuster, 2006) and competes with the migration of electron hole along the strands (Charkaborty, 2007). The experimental and electronic structure calculations (Khistyaev et al., 2013) showed that ionization-induced PT between nucleobases is endothermic in AT (neutral state) while exothermic in ionized species. The barrierless H-bonded pairs in ionization-induced species predict higher efficiency for the process. PT is slightly endothermic in π-stacked systems as no hydrogen bonding exists in the complexes. PT reactions occur via transition state (TS) (Hratchian and Schlegel, 2005) with one imaginary frequency connecting the reagent and product at the PES. Then, the reaction proceeds over or under the barrier via the tunneling (Löwdin, 1963; Bell, 1980; Koch et al., 2017; Piatkowski et al., 2018; Smedarchina et al., 2018). At this stage, the energy level of proton is equal for the reactants and products (Li et al., 2011; Ceriotti et al., 2016). The path integral molecular dynamics (PIMD) reveal that the nuclear quantum effects (NQEs) can also change the relative stability of tautomeric forms of DNA base pairs (Pérez et al., 2010). Car–Parrinello-based PIMD has been used to see the effect of NQEs during the DPT process in similar DNA structures.

Water is considered as a very important complex in the PT biological systems (Ball, 2013). For example, water filled ion channels through interfaces and membranes and in aerosols (Voth, 2006). A relay-type transport of protons is provided by water wires, which is important for all processes (Agmon, 1995; Chandler et al., 2012). Water wires are involved in the proton-coupled electron transfer in DNA. Experimental studies have been carried out for the NO+(H_2_O)_*n*_ clusters with proton-coupled water activation in the ionosphere (Relph et al., 2010). The excited state photo acid structures (Mohammed et al., 2005), proton vs. hydrogen transfer pathways, and the effect of water solvation on catalytic action on tautomers equilibria via PT (Sukhodub, 1987; Kim et al., 1996, 2007; Mizuse et al., 2011) were also studied. The experimental and energetic calculations on dimethyluracil dimers predict superficial proton transfer in the absence of hydrogen bonds (Golan et al., 2012). Experiments were carried out to investigate the nature and dynamics of proton transfer in stacked systems and molecular dynamics calculations were carried out to visualize the actual proton transfer mechanism.

**Figure 5:**
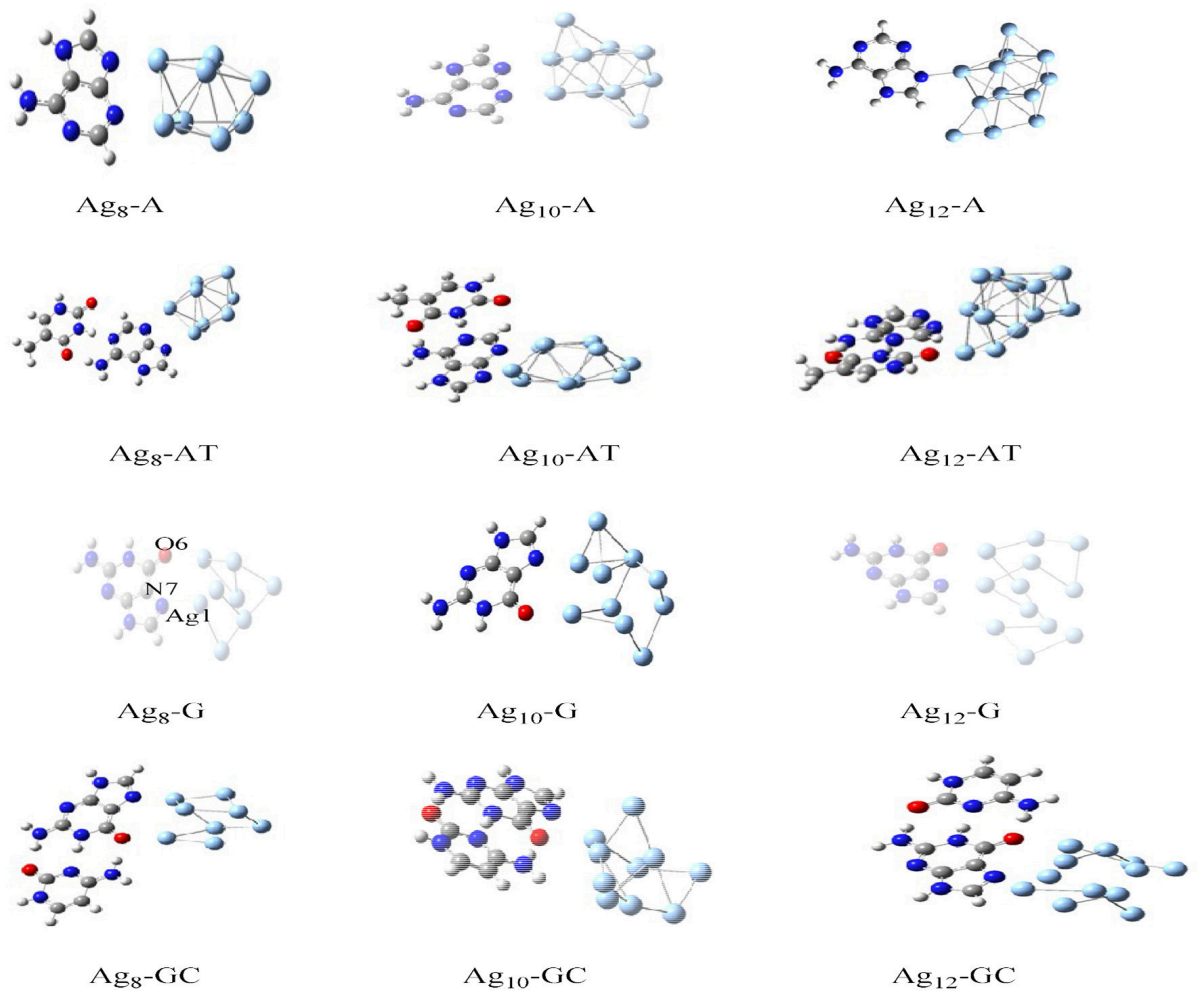
Optimized structures of Ag_8_-A, AT, G, and GC base pairs (Adapted with permission from Taylor and Francis).

**Figure 6:**
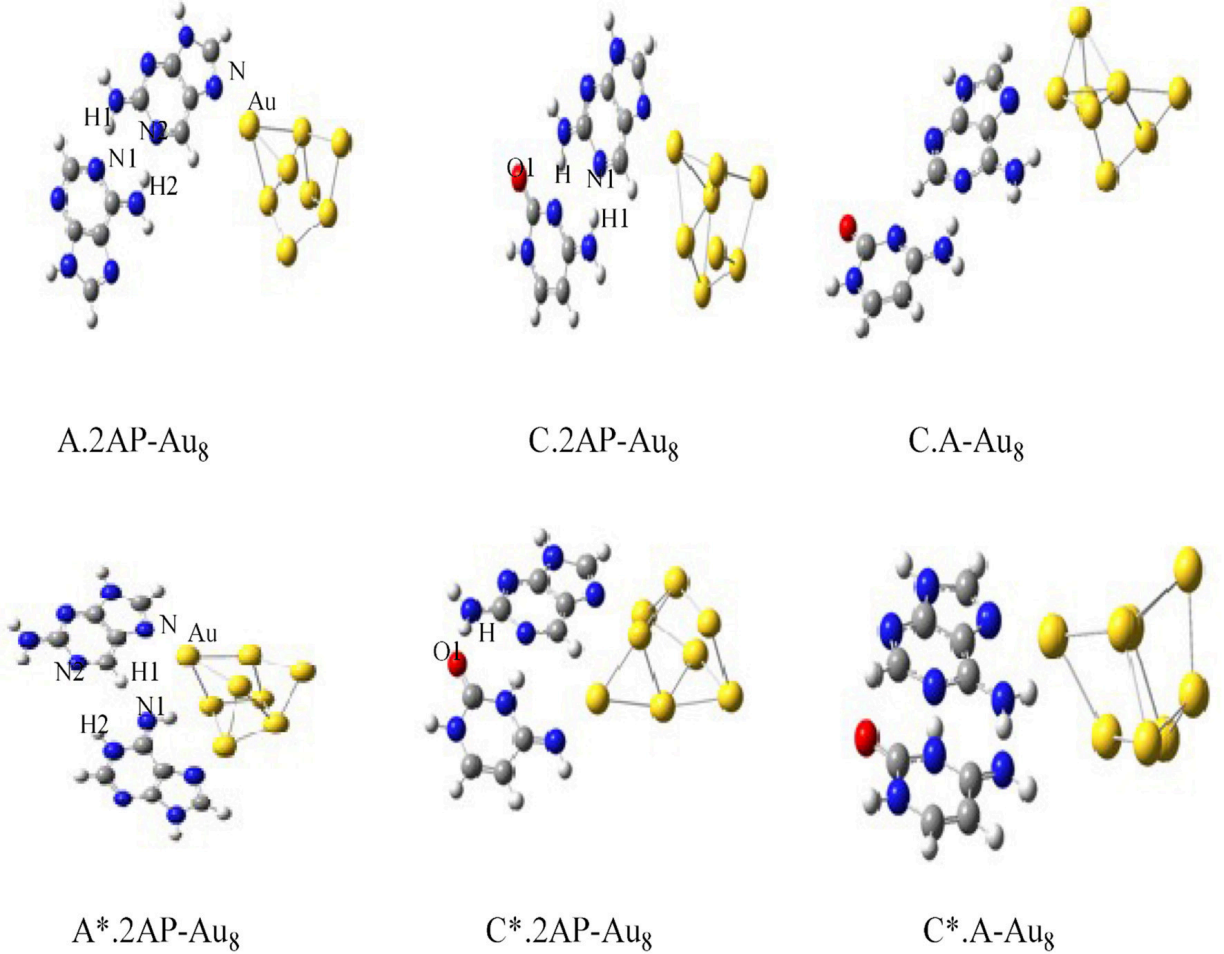
Optimized structures of [A.2AP(w)/A^*^.2AP(WC)/C.2AP(w)/C^*^.2AP(WC)/C.A(w)/C^*^.A(WC)]–Au_8_ complexes (Adapted with permission from Taylor and Francis).

The charge transport in DNA also has potential applications in molecular electronic devices, DNA damage, and repair (Seeman, 2001; Boon et al., 2003; Schuster, 2004; Holman et al., 2007; Jacquemin et al., 2014; Zhang et al., 2015). We have carried out studies on neutral silver cluster interaction to DNA bases/WC base pairs (Srivastava, 2018) using DFT with hybrid density functional B3LYP (Lee et al., 1988; Becke, 1993) potential and LANL2DZ (Hay, 2002)/6–31+G^**^ basis set. See Figure 5. The results reveal strong silver–WC pair's interaction and absorption in the visible region. Similarly, theoretical studies on the electronic and optoelectronic properties of [A.2AP(w)/A^*^0.2AP(WC)/C.2AP(w)/C^*^0.2AP(WC)/C.A(w)/C^*^.A(WC)]–Au_8_ mismatch nucleobase complexes (Srivastava, 2017) were carried out at the B3LYP/6-311++G(d,p) level. After analyzing the different parameters, the results indicated the applicability of these complexes in fluorescent bioimaging. The smaller HOMO–LUMO (HL) band gap suggested strong chances of electron transfer through DNA duplexes (see Figure 6).

**Figure 7:**
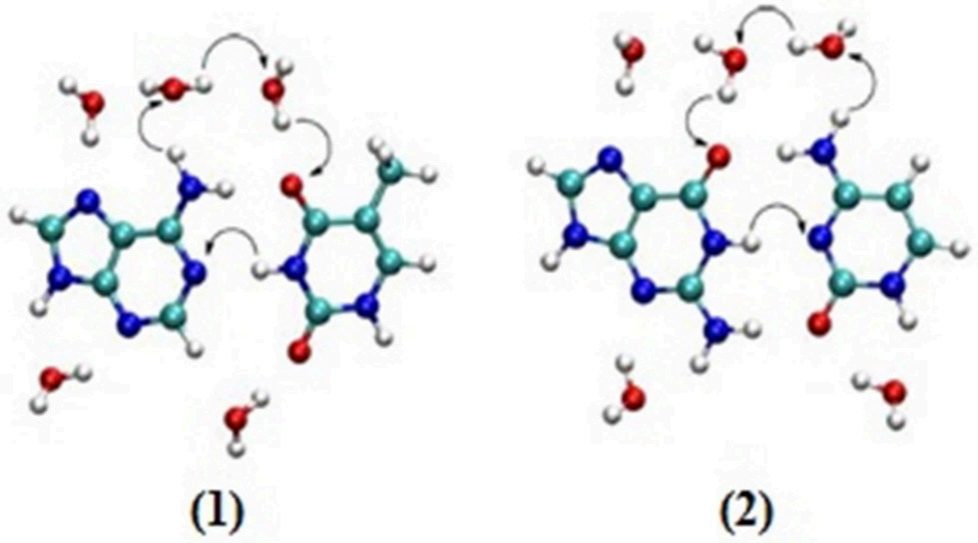
Suggested mechanisms (by arrows) for double proton transfer in hydrated (1) AT and (2) GC base pairs.

Studies have been carried out to find the relationship between charge transport and proton transfer. The effect of water molecules led to proton transfer for the cationic stack of AT while a pK_a_ experiment does not predict proton transfer in AT base pairs (Steenken, 1989; Colson et al., 1992). A recent study reported that proton transfer occurs at high temperature as well. Here, two different mechanisms (displacement and oriental polarization) were proposed for the CT and PT at different degrees of temperatures (Zengtao et al., 2019). It was observed that multiple proton transfer occurred at low temperatures and the water-assisted PT occurred by displacement polarization and oriented polarization mechanisms. The rate of PT decreased at higher temperature with water shifting the polarization mode to enhance the PT rate. Further, it was concluded that higher temperature lowers the probability of proton transfer and PT is favored at low temperatures. The DPT mechanism for hydrated AT and GC pairs is given in Figure 7.

The results for the series of nucleobase complexes with organic or inorganic proton donors indicated that electron attachment to these complexes in the gas phase induces PT, which leads to the strong stability for the valence anions (Dabkowska et al., 2004; Harańczyk et al., 2004a,b; Haranczyk et al., 2005; Radisic et al., 2005; Mazurkiewicz et al., 2007b). The probability of stable anion formation in the surrounding of DNA complexes was also studied. The intermolecular PT to excess electron attachment of adenine–formic acid hydrogen-bonded complexes indicated more involvement of adenine bases in proton-donor and -acceptor centers and hydrogen-bonding interactions (Mazurkiewicz et al., 2007a). Further, it was predicted that the stability of the valence ion will increase with the involvement of more species with the nucleobases. As the large affinities of adenine complexes are counter balanced by many physiological environmental factors, they play an important role in the induced mutations by low energy electrons.

Studies have been conducted for the selectively modified DNA structures, which show promising applications for ultrashort electric pulses in medicine. QM/MM (Schrödinger, 1945; Watson and Crick, 1953) became the perfect choice to study the condensed phase biomolecular reactions (Kryachko, 2002; Bertran et al., 2006). QM/MM approach is a reliable and sensitive computational approach for the larger systems. The effect of QM size, method, and schemes on the computed proton transfer energetics in a DNA double helix (Floriań and Leszczyński, 1996), the role of external agents on the PT tautomeric equilibria, and the mutations in electric fields were studied by QM/MM approaches. The influence of embedding and coupling schemes by the QM/MM approach (Roßbach and Ochsenfeld, 2017) was investigated for the transfer of a proton in a DNA base pair. The results indicated that the embedding scheme (mechanical or electrostatic) has much greater impact on the convergence behavior than the coupling scheme (additive QM/MM or subtractive ONIOM).

An analytical review on two-dimensional (2D) potential-energy surface based on two equal hydrogen bonds coupled by a correlation term was given to describe the dynamics of the DPT mechanism (Smedarchina et al., 2008). The quantum-mechanical tunneling and its role in chemical transformations were explained in a recent review (Meisner and Kästner, 2016). The “Tunnel effect” affects the reaction paths and branching ratios and influences the biochemical processes. In another review, a new discipline called “quantum biology” was introduced (McFadden and Al-Khalili, 2018), which included not only the parameters of quantum mechanics such as coherence, tunneling, and entanglement but also photosynthesis, enzyme catalysis, avian navigation, or olfaction. The work has been carried out for the dominant role of tunneling in condensed phases and at high temperatures for DHT in porphycenes by experimental means. These experimental results have provided deep experimental insight into the hydrogen transfer phenomena (Ciaćka et al., 2016).

**Figure 8:**
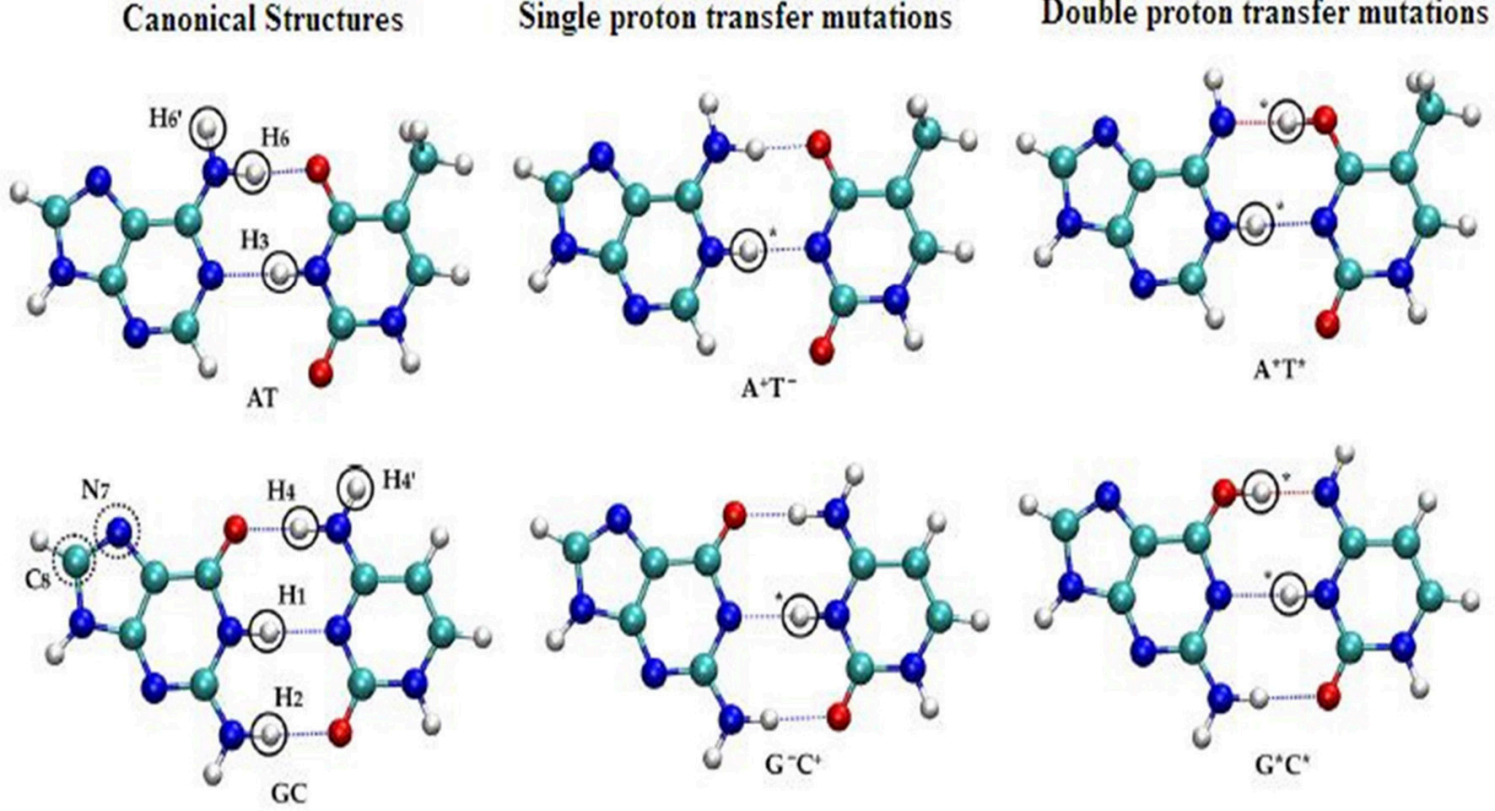
Structural representation of the single and double proton transfer mechanisms of canonical (AT, GC) pairs.

The SPT and DPT mechanisms in DNA were carried out by many refined models (Kryachko, 2002; Bertran et al., 2006; Kumar and Sevilla, 2010) using second-order Møller–Plesset (MP2) energies on Hartree–Fock (HF) geometries in WC base pairs (Floriań et al., 1994; Floriań and Leszczyński, 1996). In these studies, the mutations were energetically favorable for GC pairs. The tautomeric equilibria of isolated WC base pairs in gas phase and solvent phase (Gorb et al., 2004) were studied using the CASSCF/CASPT2 method to map the 2D PESs for the GC pair by three different mechanisms, which is stepwise double proton transfer (SDPT), DHT, and the concerted double proton transfer (CDPT). The accurate theoretical predictions for biological activities were given with the proposed chemical models. See Figure 8 for SPT and DPT in canonical WC pairs.

The effect of the surroundings on the spontaneous tautomeric mutation in DNA (Cerón-Carrasco et al., 2009a,b) showed that water molecules are responsible for the double-helix DNA architecture (Kabelác and Hobza, 2007). Catalyzing the spontaneous mutation increases the proton acceptance and donation with DNA. The SPT zwitterionic product acts as a transient species, and the shift of equilibrium to the canonical form occurred for the direct and water-assisted mechanism in AT base pair. Mutations can induce in GC only during the DPT mechanism. The spontaneous mutation for DPT was observed in a propeller-like conformation for the studies of single GC base pairs (Floriań and Leszczyński, 1996; Gorb et al., 2004). The PT reaction for GC radical anion (Chen et al., 2009) with hydrated model and stacking effects was carried out with a different level of DFT. Liang and co-workers used the molecular dynamics (MD) method to study the motions of proton (Xiao et al., 2012).

Prof. Hovorun's group has carried out studies for the canonical A·T(WC) and G·C(WC) WC complexes (Löwdin, 1963; Gorb et al., 2004; Brovarets' et al., 2012; Brovarets' and Hovorun, 2014a,b, 2015e; Roßbach and Ochsenfeld, 2017), wobble pairs Padermshoke et al., 2008; Brovarets' et al., 2015, model protein–DNA complexes (Brovarets' et al., 2012), water-assisted proton transfer in nucleosides (Markova et al., 2017), and mutagenic tautomers (Kondratyuk et al., 2000; Samijlenko et al., 2004; Platonov et al., 2005) to search the reason for the spontaneous point mutations, and other important biological functions such as heredity, aging, and diseases (Löwdin, 1963). These mutations can be considered as “quantum jump” between the undamaged chromosomes and its mutated counterpart states. PT reactions in DNA can be altered by metals and free radicals or the high-energy radiation and electric field.

Though the genotoxic agents create a damaging role in spontaneous mutations, its influence in cancer cells can be positively controlled (Xiao et al., 2012). Chemical reagents as Pt–DNA adduct support SPT with near-planar structures (Korolev et al., 2009). PT reactions also support the biological activity of cisplatin, carboplatin, and oxalaplatin for sequence-specific mutations by the effects of metal ions (Na^+^, K^+^, Ca^2+^, and Mg^2+^) (Rozsnyai and Ladik, 1970). A novel approach for the internal treatment of cancer with the action of an external physical agent with chemotherapeutic drug was suggested, which controls the exposure region and time duration. Different computational methods have been opted on small DNA models (Jissy and Datta, 2010; Arabi and Matta, 2011) and GC base pairs (Cerón-Carrasco and Jacquemin, 2013) to study the PT reactions by applying intense external electric fields.

The intramolecular PT reactions were studied for both isolated and hydrated DNA bases, while the intermolecular SP and DP transfer reactions are seen in the nucleic acid bases (dimers) at both ground and excited electronic states (Sekiya and Sakota, 2008). Small activation barriers were seen for GC (anionic and cationic) pairs. The tautomeric equilibria and kinetic parameters (lifetime and rate constants) were defined by the activation barrier (Atkins, 1998).

Further, it was studied that the PT reaction is more favorable for the anion as compared to the cation. In the uracil and alanine interactions, the results demonstrated the possibility of electron-induced mutations in DNA–protein complexes. Neutral radicals of hydrogenated pyrimidine nucleobases also lead to mutations in DNA and RNA by low-energy electrons (Beierlein et al., 2011). The effect of polarity was also studied for the dipole-bound anionic states.

DNA replication errors that are caused by genomic instability (Liu et al., 2014; Tomasetti et al., 2017) occurred by the mutagenic tautomerization of WC pairs (Watson and Crick, 1953). The high-energy tautomerized states (Brovarets' and Hovorun, 2010, 2015f) and wobble pairs (Brovarets' and Hovorun, 2015b) for the mutagenic tautomerization of canonical base pairs were studied by Prof. Brovarets et al. The reverse barrier is absent in the tautomeric A·T(WC) pair of DNA bases and have a small kT value for the G·C(WC) DNA base pair (Gorb et al., 2004; Bertran et al., 2006; Brovarets' et al., 2012; Brovarets' and Hovorun, 2014a, 2015a), indicating the non-applicability of Löwdin theory of DPT. The tautomerization occurred via the sequential intrapair proton transfer and shifted to the related pairs (Brovarets' and Hovorun, 2015b). The studies were followed by other similar studies (Brovarets' and Hovorun, 2009, 2015b,c,d, 2016) by the same group. Results indicated that the non-dissociative transitions occurred via specific intermolecular interaction along the intrinsic reaction coordinate (IRC) (Brovarets' and Pérez-Sánchez, 2017). It showed that the tautomeric transitions for A·T DNA base pairs are non-planar and high-energetic intrapair bases (Brovarets' and Hovorun, 2014a, 2015b,d; Brovarets' et al., 2015, 2018a,b,c,d).

Cytosine plays a crucial role in DNA/RNA base pairing as several hydrogen-bonding patterns due to involvement in the genetic codon of 17 amino acids. Interestingly, the proton transfer reactions for cytosine-specific DNA-binding proteins for cytosine and 5-fluorocytosine with 5-nitrouracil, CytNit, and 5FcytNit were also studied (Portalone, 2011).

The strength and the directional properties of non-covalent binding interactions (hydrogen bonding, ionic interactions, van der Waals, and π-π stacking) were used to design the self-assembled or self-associated compounds (Watson and Crick, 1953; Dahm, 2008). The hydrogen-bonding association results in complete proton transfer for an ionic compound with the reinforcement of non-covalent interactions (Merchán and Serrano-Andrés, 2003). The rare tautomeric forms were produced by the intermolecular single/double proton transfer (SPT/DPT) reactions in the hydrogen-bonding DNA pairs. For the unusual combination of NABs pair, normal hydrogen-bonding pattern in DNA is altered, which leads to spontaneous mutations. The DPT reaction is more favorable than the SPT for the neutral systems. The SPT products for base pairs are largely stabilized due to the transfer of a positive charge. The output products are stable and involved in the mutagenic processes. SPT occurs in the formation of the ion-pair complex, while the DPT process retains the electroneutrality. In DPT, the energy barrier is high with the thermodynamically unstable double tautomers. DPT reaction does not show any mutagenic effects. The structural mechanisms for spontaneous point mutations (Turaeva and Brown-Kennerly, 2015) in DNA occur through the transitions and transversions, and various enzymatic complexes for DNA lesions are involved in the repair process, such as DNA glycolases, polymerases, and photolyases (Fromme et al., 2004; Braithwaite et al., 2005). The stable canonical NAB pairs were studied to understand the mechanism of genetic code (Blancafort et al., 2005; Chen and Li, 2005; Marian, 2005; Perun et al., 2005; Tomić et al., 2005). The protonation of NABs also contributes to the stabilization of triple helix, which is stabilized at acidic pH. The PT reaction is also seen in the molecular identification of cytosine–acidic nucleobase derivatives (Park et al., 2011). The DPT in prototropic tautomerisms for many amidine systems and porphyrins was studied experimentally, and the rates and the kinetic isotope effects for both types of DPT were reported. The interaction of amidines with carboxylic acids is useful as the guanidine moiety serves as the binding site for carboxylic acids.

Twenty-two biologically important nucleotide base pairs were studied (Brovarets' and Hovorun, 2010, 2015f) to understand the microstructural mechanisms via DPT and intermolecular hydrogen bonding. It was predicted that the short-lived, low-populated mispairs provide long-lived A·C^*^ (Brovarets' and Hovorun, 2015a), G^*^·T (Brovarets' and Hovorun, 2015b,c), and H·T^*^ base pairs. The DPT tautomerizations are dipole active and depend on the orientation and change in dipole moment. These complexes can be used for molecular generators under external electric fields (Cerón-Carrasco and Jacquemin, 2013; Mandal et al., 2015; Zhang et al., 2015; Ruiz-Blanco et al., 2017). Further reactive forces via IRC were studied for the reactant, transition states, and product. The cooperativity of the specific intermolecular interactions, i.e., non-classical bonds, loosened covalent bridges, and attractive van der Waals contacts were also analyzed.

**Figure 9:**
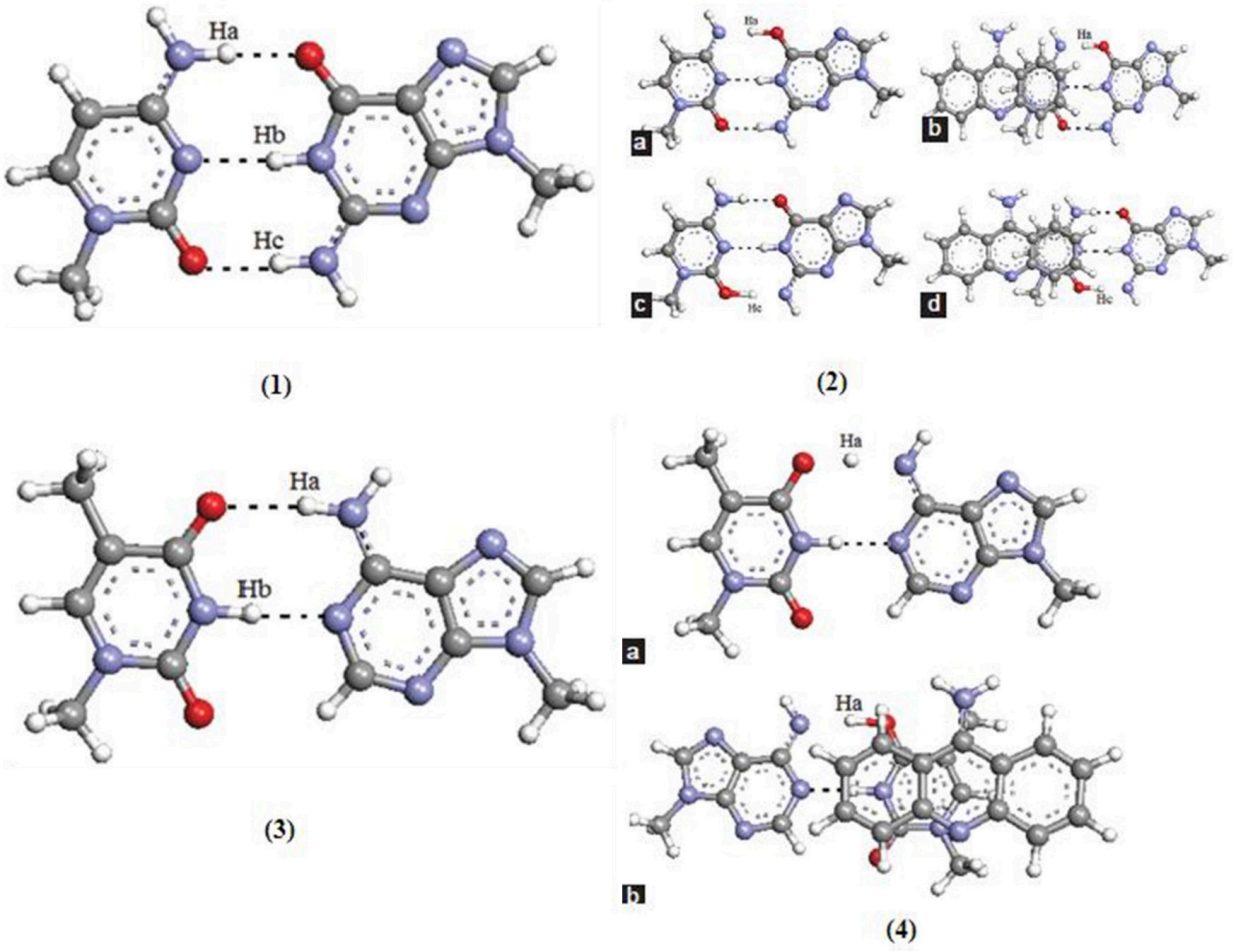
Optimized structure of **(1)** guanine–cytosine base pair; transition states of **(2a)** unstacked GC base pair for proton (Ha) transfer type PT1, **(2b)** stacked GC base pair for proton (Ha) transfer type PT1, **(2c)** unstacked GC base pair for proton (Hc) transfer type PT2, and **(2d)** stacked GC base pair for proton (Hc) transfer type PT2; **(3)** optimized structure of (3) adenine–thymine base pair; transition states of **(4a)** unstacked GC base pair for proton (Ha) transfer type PT1, **(4b)** stacked GC base pair for proton (Ha) transfer type PT1 (Adapted from Bezbaruah and Medhi, 2016).

New pathways for the mutagenic tautomerization of the classical A.T DNA base pairs in free state, canonical A.T Watson-Crick (WC), reverse A.T Watson-Crick (rWC), A.T Hoogsteen (H), and reverse A.T Hoogsteen (rH) pairs were studied via a sequential PT method. The WC base pairs consist of GC and AT base pairs, while the Hoogsteen base pair is a variation of base-pairing in nucleic acids, such as A•T base pair. The two nucleobases, one on each strand, can be held together by hydrogen bonds in the major groove. A Hoogsteen base pair applies the N7 position of the purine base and C6 amino group to bind the WC (N3–C4) face of the pyrimidine base. The results revealed significant changes in the mutual orientation of the base pairs. Various factors were studied for the process, such as transition states, symmetry, tight ion pairs, Gibbs free energies of activation, and the tautomeric transitions. It was observed that the product of the tautomerization of these pairs was transformed into the energetically favorable [A.T^*^(rwWC), A.T^*^(rwH), and A.T^*^O_2_(wH)] wobble mispairs through DPT. In these PT processes, the DNA bases shift laterally relative to each other, leading to the wobble pairs of mutagenic tautomers (Brovarets' et al., 2015). This mechanism was applied for the mutagenic tautomerization of various other mispairs (Brovarets' and Hovorun, 2009, 2015b,c,d; Brovarets' et al., 2018e). The mechanism does not provide sufficient long-lived mutagenic DNA tautomers and escape from the replicative DNA-pol, transforming into the canonical tautomeric forms. The optimized structures and the transition states of AT and GC base pairs are given in Figure 9.

GC radical cation base pair was studied theoretically by the use of DFT, B3LYP/6-31+G^**^ calculations (Kumar and Sevilla, 2009) to analyze the role of hydration on PT mechanism. In GC pairs, holes and electrons were created due to direct interaction of ionizing radiation. Holes were initially captured by guanine (lowest oxidation potential), while thymine and cytosine have captured the electron due to higher electron affinities. Recent studies indicated that hydration has a pronounced effect on the experimental results, and it favors the proton transfer reaction within the base pairs (Adhikary et al., 2006; Witwicki et al., 2009). The effect of bulk solvent is negligible in fully hydrated equilibrium and the stability of the deprotonated states depend on the interactions of water molecules (Lill and Helms, 2001). The energy surface of intermolecular proton transfers can also be reproduced when the assisted water molecules are replaced by point charges. The charges need to be enhanced at close distances due to the induced polarization.

Latest reports included the bystander effects (damage created by irradiated cells in the environment) to study the untargeted mutations (Whiteside et al., 2011). Bystander effects are basically the combination of untargeted and delayed mutations and can be studied experimentally. Untargeted base substitution mutations include some nucleotides inserted to the undamaged sites of DNA. Five rare tautomeric forms of AT pairs within the neighborhood from cyclobutane dimers were studied during DNA synthesis (Niwa, 2006). It was predicted that radiation itself induces genomic instability in cells, which cause genetic changes and increase the rate of mutations. Genomic instability occurs due to the delayed mutations and untargeted mutations that lead to cancer. Six mechanistic explanations were provided to explain the bystander effects. The most probable explanation is the occurrence of non-specific molecular damage due to an irreversible regulatory change in the dynamic interaction network of the cellular gene products (Averbeck, 2010; Campa et al., 2015). Though the conventional reason for mutations depends on sporadic errors of DNA polymerases (Taylor, 2002; Wright, 2010; Watanabe, 2015), one more explanation is given by A-rule, in which the non-complimentary bases are inserted to the DNA polymerases against the damages (Wright, 2010; Watanabe, 2015). Still, there remain many unanswered questions related to the untargeted mutations. For example, why do mutations occur on some undamaged sites of DNA? What is the difference between the undamaged and untargeted mutation sites? Why does genomic instability increase in an unnatural manner with the number of untargeted and delayed mutations? Which external agent is responsible to selectively damage DNA, leaving the non-target sites?

The explanation was given by the polymerase-tautomeric model, which is used to explain the formation of targeted (base substitution, insertions, and deletions) mutations and UV-induced mutagenesis. Also, the polymerase-tautomeric model for bystander effects explains the mechanism for the formation of delayed targeted and untargeted base substitution mutations.

The interactions of anticancer drugs to the DNA nucleobases were studied by the proton transfer mechanism (Bezbaruah and Medhi, 2016). The interaction was carried out for normal base pairs and drug–base pair stacked models. The stacking interactions of drugs–DNA bases results in change in PT energies. PT may also change the acid–base characteristics during the process. The changes in barrier of proton transfer and shifting of equilibrium were also seen in these drugs–DNA bases interactions.

DNA and RNA are both essential for the living organism. DNA keeps the hereditary information of the cell whereas RNA contains the necessary codes to convert that information into functional products. Both RNA and DNA duplexes have large sugar–phosphate chains. The polymerization of amino acids forms the messenger RNA (mRNA) (Sprinzl, 2006). Translational adaptor recognizes the position of the mRNA and the particular place of amino acid is fulfilled by the transfer RNA (tRNA). Each of the other 40 different tRNAs has two specific functions: (i) recognition of its anticodon triplet, a three-letter code of the mRNA, and (ii) the tRNA accepts the incoming amino acid on its chemical site of 3′-terminal adenosine and carries it to the A-site of peptidyl transferees for the formation of peptide bond at the ribosomal center. The post-transcriptional modification of nucleotides in RNA occurs as tRNA, rRNA, mRNA, snRNA, snoRNA, and tmRNA. In this way, 96 different types of nucleotides are identified till now (Rozenski et al., 1999). Other suggested modifications were methylation, pseudouridylation, and thiouridylation, which occurred earlier (Sloan et al., 2017). The role of modified transitions from RNA to DNA was suggested by Martínez Giménez et al. (1998). Thirty-five modifications were found in the peptidyl transferase center, and the A, P, and E sites of tRNA, mRNA, the polypeptide tunnel, and subunit interface. Though these modifications are not essential for ribosomal functioning, they improve the efficiency for ribosomes at a global level (Decatur and Fournier, 2002). The folding and unfolding of hairpin ribozyme have multiple conformations of the RNA with distinct kinetics, and it was studied that the mechanical stability of RNA pseudoknots has strong correlation with their frame shifting efficiency during translation. RNA species hairpin (Li and Tinoco, 2009), pseudoknot (Chen et al., 2007), ribozyme (Onoa et al., 2003), kissing complex (Li and Tinoco, 2009), and riboswitch (Greenleaf et al., 2008) were also studied. The kinetics of reactions was measured by the lifetimes of each species in the reaction. The change in Gibbs was obtained from the mechanical work done (reversible). The reversibility and the unreversibility of the unfolding/folding reaction were measured by the occurrence of the same force in the forward process and no hysteresis, respectively. However, the reversible work for non-reversible reaction was obtained from the distribution of non-reversible work values.

RNA is a four-helix-junction structure (self-cleaving), which contains two internal loops (Wilson et al., 2005). RNA helicases use nucleotide triphosphates (NTPs) to unwind RNA duplexes and are involved in many RNA metabolism processes (Jankowsky and Fairman, 2007; Pyle, 2008). The hairpin ribozyme was derived from tobacco ring spot virus satellite. Investigations were carried out for the role of proton transfer using kinetic parameters in the catalytic mechanism of polyadenylate polymerase (PAP) for the forward (adenylyl transfer) and reverse (pyrophosphorolysis) reactions. Forward reaction suggested the involvement of two protonic species. The multi-conformational continuum electrostatic (MCCE) method was performed to calculate the kinetic parameters and pH variation. Results indicated that PAP contains three globular domains and adopts a closed, active conformation (Balbo and Bohm, 2007; Balbo et al., 2007) and (Mg^2+^) ion mechanism (Bard et al., 2000; Martin et al., 2000).

The involvement of proton transfer in the ribozyme mechanism is observed in the past few years. Proton transfer is known as general/specific acid–base catalysis (Jencks, 1969; Silverman, 2000) when it occurs to/from biopolymer side chains, water, or OH and hydronium ions, respectively. Acid–base chemistry is observed in every enzyme-catalyzed chemical reaction, except the radical reactions. In ribozyme reactions, the PT prevents the accumulation of unfavorable intermediates that bear charge on bridging oxygen atoms. In general acid–base catalysis, the charge does not accumulate on the 2′- or 5′-oxygens. To optimize general acid–base chemistry, RNA uses the pK_a_ shifting mechanism. In class 1 method, the loaded proton is sequestered in hydrogen bonding, for example, RNA and DNA helices (Wang et al., 1997; Allawi and Santa Lucia, 1998; Pan et al., 1999), cleavage site of the hairpin ribozyme (Cai and Tinoco, 1996; Butcher et al., 1999), lead-dependent ribozyme (Legault and Pardi, 1994), protonated adenine in a Hoogsteen interaction with guanine in a DNA duplex (Gao and Patel, 1988; Leonard et al., 1990; Carbonnaux et al., 1991), protonated cytosine forming a wobble base pair with guanine in the tetrahymena ribozyme (Knitt et al., 1994), protonated cytosine–cytosine base pair (Borah and Wood, 1976), protonated cytosine forming a Hoogsteen pair, and DNA–antibiotic (Quigley et al, 1986) complexes. In class 2, proton is exposed, for example, a model for the transition state of the human hepatitis D virus (HDV) ribozyme self-cleavage reaction (Nakano et al., 2000), Hoogsteen–Hoogsteen interactions for two protonated adenines (Rich et al., 1961), adenine and protonated adenine in a DNA duplex (Chou et al., 1992; Maskos et al., 1993), and guanine and protonated adenine in a Hoogsteen pairing. In RNA folding, co-operativity acts as a driving force in pKa shifting (Moody et al., 2005). Larger interactions lead to bigger pKa shift. Further proton transfer in five different small ribozymes, Hepatitis Delta Virus Ribozyme, Hairpin Ribozyme, Hammerhead Ribozyme, VS Ribozyme, and glmS Ribozyme, were studied with novel biochemical experiments and theoretical means to understand the mechanism. Computational studies have been carried out for the charged nucleobases in RNA, which indicated the thermodynamically unstable ionized nucleobases at pH ~7.4 (Halder et al., 2019). Water affects the systems through hydrogen bonding, Coulomb interactions, and the mediation of proton transfer as an intrinsic component in the secondary structure of proteins.

The catalytic role for a conserved adenosine residue in 23S RNA of the large subunit of ribosome was proposed from the experimental crystallographic and chemical modification methods (Ban et al., 2000; Muth et al., 2000; Nissen et al., 2000). The RNA side chains were not suited for acid–base catalysis as the pKa values of these side chains have significantly higher or lower values than neutral pH. Thus, a significant shift in pKa will be required.

Interestingly, the nucleic acid bases (NABs) with sulfur atom were also studied in natural tRNAs (Yekeler, 2000). These pairs are useful in many biological activities such as thyroid-regulating activities and antitumor agents and in numerous metabolic processes. The effect of sulfur atom on the conformation of the RNA helix was studied as these thiouracil derivatives are useful for the treatment of anti-HIV (Al-Masoudi et al., 2011), cardiovascular diseases (Ribeiro Da Silva et al., 2013), and others (Wen et al., 2006). Various mechanisms were suggested to study these structures as the effect on 2-thiouridine on the RNA structure remains unclear (Sochacka et al., 2015). The DFT studies show lower stability of these base pairs with sulfur and the retard/deform shape of the strand with the RNA viral/tumoral, used for the pharmaceutical reagents. It also gives predictive insight into the structural and energetic effects of these nucleobase modifications, such as post-transcriptional changes, ionization, tautomerization, and metal ion coordination to the nucleobases.

## Conclusions

Proton transfer reactions are a very important catalytic activity to control virtually all chemical reactions. Proton shuttles are used in the transfer mechanism of ribosomes and large ribozymes. Even after 50 years of Löwdin's hypothesis, the mechanism behind the rare tautomeric forms in DNA remains unclear. However, quantum computational calculation has accredited several research groups to traverse proton transfer reactions in DNA. Recently, investigations were carried out for the possible modifications of natural tautomeric equilibria, which are induced by external agents. In DNA base pairs, GC base pair emerged as the foundation of DNA replication. It clearly bound the double strands and promotes mutations through induced PT reactions either by chemical or by physical agents. There is a least but finite probability for protons to change place within the hydrogen bond due to quantum tunneling, which will alter the genetic code and cause mutations. These mutations could be the cause of several diseases such as cancer. As the PT reactions occurring in DNA base pairs are explored now, the progress in the control of the spontaneous mutation mechanism is anticipated in the future, which will manage to control the suicide of malignant cells. Water-mediated interactions, altering of energy levels in solvated species, and modified PES along reaction coordinates facilitate effective proton transport through the interfaces and ion channels, which need to be explored.

The regulation of proton tunneling has opened a new area of research in bioelectronics and biocomputing as well. This effect is applied as a “control gate in biocomputing” and realized by a large number of enzymatic reactions. As the tunneling in semiconductors is sensitive to their environment, the devices can be used as sensors. The enzyme tunneling of protons is sensitive to the temperature, external pressure, and others, so focus should be toward the studies of external parameters such as the magnetic field, electromagnetic wave frequency and intensity, etc., which will open new prospects in the external control and information transfer in bioelectronics.

## Author Contributions

The author confirms being the sole contributor of this work and has approved it for publication.

### Conflict of Interest Statement

The author declares that the research was conducted in the absence of any commercial or financial relationships that could be construed as a potential conflict of interest.
